# Catastrophic ENT Complications of Pediatric Infectious Mononucleosis: Recent Experience at a Tertiary Pediatric Hospital

**DOI:** 10.3390/jcm15072516

**Published:** 2026-03-25

**Authors:** Lorenzo Gaini, Anna Cozzi, Gioia Piatti, Michele Gaffuri, Samantha Bosis, Paola Marchisio, Giovanna Ghidini, Giorgio Croci, Antonio Carpino, Sara Torretta

**Affiliations:** 1 Fondazione IRCCS Cà Granda Ospedale Maggiore Policlinico di Milano, 20122 Milano, Italy; lorenzo.gaini@unimi.it (L.G.); anna.cozzi@unimi.it (A.C.); michele.gaffuri@unimi.it (M.G.); samantha.bosis@policlinico.mi.it (S.B.); paola.marchisio@unimi.it (P.M.); giovanna.ghidini@policlinico.mi.it (G.G.); giorgio.croci@unimi.it (G.C.); antonio.carpino@policlinico.mi.it (A.C.); 2 Department of Clinical Sciences and Community Health, University of Milan, 20122 Milano, Italy; gioia.piatti@unimi.it

**Keywords:** tonsillitis, EBV, mononucleosis, children, airway obstruction

## Abstract

**Background:** Epstein-Barr virus-related acute pharyngotonsillitis is common in children and adolescents and is generally managed successfully with positive outcomes by both ENT specialists and pediatricians. However, a variety of acute, life-threatening complications can occur, including upper airway obstruction and infectious or immune-mediated sequelae. **Methods:** This paper describes our recent experience with four pediatric patients presenting with severe ENT manifestations of infectious mononucleosis (IM) that led to life-threatening complications, all of whom were hospitalized and managed at our tertiary pediatric hospital between January 2022 and April 2025. **Results:** We report four cases (two boys and two girls) aged 5 to 16 years, hospitalized with complicated EBV-related pharyngotonsillitis. Presentations included respiratory distress (cases 1 and 2), hemophagocytic lymphohistiocytosis resulting in death (case 3), and a retropharyngeal abscess (case 4). **Conclusions:** The prognosis of IM in the pediatric population is generally favourable. However, acute, life-threatening complications may arise. In such cases, timely and coordinated multidisciplinary management involving ENT specialists, pediatricians, and anesthesiologists is crucial.

## 1. Introduction

Infectious mononucleosis (IM) is an acute viral infection that most commonly affects children and young adults, with an incidence of approximately 6 to 8 cases per 1000 person-years [1].

Epstein-Barr virus (EBV), a double-stranded DNA virus of the Herpesviridae family, is the primary cause of IM, accounting for approximately 90% of cases. A minority of cases (about 10%) are attributed to other viruses, including cytomegalovirus (CMV), human herpesvirus 6 (HHV-6), herpes simplex virus type 1 (HSV-1), and human immunodeficiency virus (HIV) [2].

The primary mode of disease transmission is through close personal contact with an infected person, particularly via saliva. It is estimated that more than 98% of the world’s adult population has been infected with EBV [3].

Infectious clinical manifestations occur when a high viral load accumulates in the palatine tonsils and adenoids, leading to acute exudative pharyngotonsillitis and adenoiditis, characterized by lymphoid hyperplasia and exudative debris. EBV infects epithelial cells and resting B cells in the oropharynx, begins replicating, and subsequently spreads throughout the body. This process corresponds to the incubation period, which lasts about six weeks and results in the activation of cytotoxic T lymphocytes and natural killer cells.

There is currently no specific treatment for IM; management is supportive, with monitoring for potential complications. Most cases are associated with a favourable prognosis. In more symptomatic cases, where high fever and pain lead to reduced intake of fluids and food, patients are often admitted to hospital—frequently to an ENT unit—for hydration, analgesia, and antipyretic treatment. Antibiotics are generally not required unless there is a confirmed bacterial superinfection.

IM typically presents with fever, acute pharyngotonsillitis, and constitutional symptoms related to the involvement of lymph nodes, liver, and spleen. It is often accompanied by hematologic abnormalities such as lymphocytosis.

One of the most frequent presenting symptoms is sore throat, which often leads to consultation with pediatricians or ear-nose-throat (ENT) specialists. Most cases are self-limiting and carry an excellent prognosis; however, in rare instances, acute complications may occur, including splenic rupture, hepatitis, and airway obstruction due to tonsillar hypertrophy. Tonsillar enlargement can sometimes lead to severe snoring and restless sleep, accompanied by respiratory pauses that may progress to hypopnea or obstructive sleep apnea. Airway obstruction occurs more frequently in younger children and is one of the most common indications for hospitalization [4]. Nevertheless, progressive airway obstruction develops in less than 5% of patients [5].

Most patients with significant upper airway obstruction can be effectively managed with clinical observation, continuous pulse oximetry, humidification, and systemic corticosteroids.

If persistent upper airway obstruction is due to severe tonsillar hyperplasia, surgical intervention should be considered. In such cases, tonsillectomy—generally performed in conjunction with adenoidectomy—should be scheduled as soon as possible [6]. In fewer than 1% of cases, if upper airway obstruction worsens and a “cannot intubate, cannot oxygenate” scenario arises, an emergency tracheotomy may be required. Tracheal obstruction leading to respiratory failure is the most common cause of death and the most lethal complication of IM [7].

In some patients, a Streptococcus pyogenes (Group A Streptococcus, or Strep A) infection can overlap with EBV-related pharyngotonsillitis. In such cases, the characteristic lymphocytosis with atypical lymphocytes observed in a complete blood count is replaced by neutrophilia, distinguishing the condition as a bacterial infection requiring appropriate antibiotic treatment. EBV impairs bacterial defence mechanisms within the tonsillar niche by depleting local IgG and secretory IgA levels, thereby increasing susceptibility to secondary bacterial superinfection, which may subsequently progress to other complications, such as peritonsillar abscess formation [8].

Beyond local complications, EBV-related acute pharyngotonsillitis—which is commonly managed by ENT specialists and pediatricians—may occasionally lead to a range of potentially life-threatening neurologic, hematologic, and systemic sequelae. These complications should be promptly recognized and managed, including by ENT physicians. According to Jenson [5], these complications include splenic or liver rupture (<0.5%); neurological complications (1–5%) such as encephalitis or meningoencephalitis, seizure, cerebellitis, cranial nerve neuritis, and Guillain-Barré syndrome; hematologic complications including hemolytic anemia (3%), thrombocytopenia (25–50%), and neutropenia (50–80%); hepatic involvement, such as hepatitis or asymptomatic elevation of transaminases (50–80%); respiratory complications, including interstitial pneumonia and pleuritis; cardiac complications, such as myocarditis and pericarditis; musculoskeletal complications, including rhabdomyolysis; and neuropsychiatric complications, including psychosis and other psychiatric disorders.

## 2. Aim of the Reported Cases

The article aims to describe cases of EBV-related pharyngotonsillitis associated with life-threatening sequelae (as reported in the Introduction section, including, in particular, respiratory distress, suppurative–septic complications, and severe alterations of the hematolymphopoietic system) occurring in pediatric patients hospitalized for severe IM in our pediatric or ENT departments between January 2022 and April 2025. Cases were defined as severe when associated with significant systemic involvement and deterioration of general clinical condition, including dehydration secondary to complete dysphagia, requiring hospitalization.

Here, we describe in detail the clinical course of four selected pediatric patients with life-threatening ENT manifestations of IM which led to severe complications, among 35 children admitted to our pediatric or ENT departments for severe IM during the study period. We specifically focus on the clinical management of patients with the most severe disease, associated with life-threatening complications, including respiratory distress, septic systemic involvement, and deep neck space abscesses.

Our case series includes two boys and two girls, aged between 5 and 16 years, who were hospitalized and presented with complicated EBV-related pharyngotonsillitis associated with respiratory distress (cases 1 and 2), hemophagocytic lymphohistiocytosis resulting in death (case 3), and a retropharyngeal abscess (case 4).

These cases were managed at a tertiary-level pediatric hospital by multidisciplinary clinical teams, including ENT specialists, pediatricians, infectious disease specialists, anesthesiologists, and intensive care unit physicians. The aim of these case reports is to highlight the potential for unexpected complications associated with IM and to emphasize the importance of multidisciplinary collaboration in patient management.

Table 1 summarizes the data of the four cases described, with particular attention to age, complications, interventions, and outcomes. All data were collected from the electronic medical records of Fondazione IRCCS Ca’ Granda Ospedale Maggiore Policlinico, Milan, Italy.

### 2.1. Case Report 1

A 14-year-old female, previously in good health, presented to our Pediatric Emergency Department (pED) in October 2022 with pharyngodynia and fever, following a first-line antibiotic treatment with cefixime. On ENT evaluation, the patient was eupnoeic but exhibited stomatolalia due to grade IV tonsillar hypertrophy, with the tonsils covered in purulent, caseous exudates. She also reported dysphagia and progressive airway compromise, without objective dyspnea at rest, but with noticeable difficulty during speech. Hospitalization was deemed necessary to initiate supportive therapy, including corticosteroids, antipyretics, and parenteral nutrition. The clinical suspicion of EBV infection was confirmed by elevated EBV viral capsid antigen (VCA) IgM levels (>160 U/mL; positivity threshold > 40 U/mL—LIAISON^®^ assay, CLIA method; DiaSorin, Saluggia, Italy). Additional relevant laboratory findings included leucocytosis with lymphomonocytosis, elevated aspartate transaminase (AST, 117 U/L), alanine aminotransferase (ALT, 130 U/L), gamma-glutamyl transferase (GGT, 110 U/L), and lactate dehydrogenase (LDH, 590 U/L).

During hospitalization in the pediatric ward, a neck and complete abdominal ultrasound were planned to rule out possible suppurative cervical lymphadenitis, as well as involvement of the liver, spleen, or abdominal lymph nodes. However, during the examination, the patient experienced a cardio-circulatory arrest, likely of hypoxic origin due to massive tonsillar hypertrophy causing oropharyngeal collapse in the supine position. Anesthesiologists were immediately alerted and performed an emergency intubation, which was successful on the second attempt. Cardiopulmonary resuscitation was initiated and successfully completed, after which the patient was transferred to our Pediatric Intensive Care Unit (pICU). A subsequent ENT evaluation revealed that the entire oro- and hypopharyngeal space was occupied by severely hypertrophic tonsils with a necrotic-hemorrhagic appearance.

**Figure 1:**
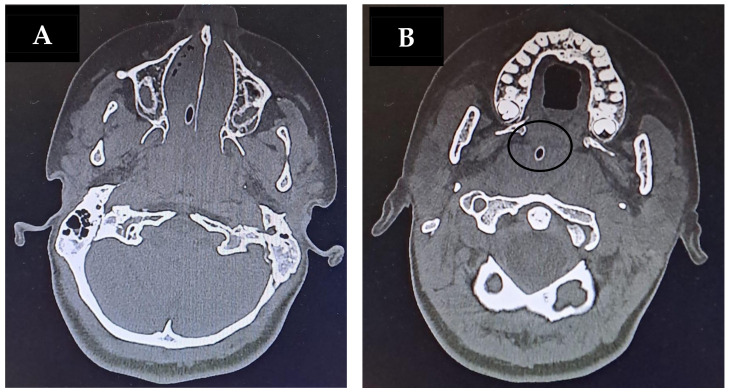
Case 1. Axial CT scan showing obstruction of the pharyngeal lumen at the upper (**A**) and lower (**B**) third of the oropharynx due to tonsillar hypertrophy. The circle highlights the nasogastric tube, compressed by hypertrophic tonsillar tissue.

After clinical stabilization and recovery of vital signs, a computed tomography (CT) scan of the neck (Figure 1), chest, abdomen, and brain was performed. The imaging revealed marked adenotonsillar hypertrophy and multiple reactive cervical lymphadenopathies, with no acute pathological findings at the brain level.

A few days later, due to persistent tonsillar hypertrophy that rendered extubation impossible despite maximal corticosteroid therapy, the patient underwent bilateral microdebrider-assisted intracapsular tonsillotomy. Histopathological examination of the tonsillar tissue confirmed benign reactive lymphoid hyperplasia, with no evidence suggestive of lymphoproliferative disorders.

The patient was successfully extubated on postoperative day two. However, due to persistent neurological slowing and aphasia of uncertain origin, a brain MRI was performed, which showed no significant abnormalities. A gradual improvement in neurological status was observed, although aphasia persisted, characterized by coherent thought but impaired speech production.

Due to significant bleeding from both tonsillar lodges on the second day after surgery, a surgical revision was required; a right extracapsular tonsillectomy and a revision of the left intracapsular tonsillectomy were performed. The following day, another bleeding episode occurred, necessitating urgent intubation and subsequent surgical revision. Coagulation tests were normal, and second-level immunological screening was negative. Upon extubation, neurological evaluation revealed dysarthria, dysphonia, and a mild gait disturbance, which progressively improved during the hospital stay.

Repeated neurological and psychiatric assessments ruled out hypoxic brain injury and suggested post-traumatic stress disorder. Additionally, during hospitalization, anxious-panic traits and a specific learning disability were identified, and the patient was referred to a psychotherapy center.

The patient underwent an extensive rehabilitation program, including behavioral therapy, speech therapy, and physiotherapy, achieving full recovery nine months after hospital discharge.

### 2.2. Case Report 2

A five-year-old, otherwise healthy, male presented to our pED in July 2023 with progressive dyspnea and deterioration of general clinical condition during an episode of acute pharyngotonsillitis. He had been receiving treatment for two days with amoxicillin at 50 mg/kg/day for fever and sore throat, following a positive rapid swab for Group A beta-hemolytic streptococcus (GABHS). On clinical evaluation, the patient exhibited evident dyspnea with retractions and sudden O_2_ desaturation, necessitating emergency intubation and transfer to the pICU.

Initial ENT evaluation, performed while the patient was intubated, revealed grade II tonsillar hypertrophy with a friable, white-coated exudate and multiple bilateral non-fluctuant cervical lymphadenopathies. Flexible nasopharyngeal endoscopy showed non-obstructive adenoidal hypertrophy.

Laboratory testing confirmed EBV infection, with a high viral load (97,393 EBV copies/mL in blood and 340,000 copies/mL from the tonsillar swab; positivity thresholds: >250 copies/mL for respiratory samples and >36 copies/mL for blood samples), measured using the ANCHOR real-time qPCR diagnostic kit (Hamburg, Germany), and a positive EBV serology (EBV-VCA IgM 129 U/mL). Additional findings included lymphomonocytosis, elevated liver function tests, and a mildly increased CRP level (1.20 mg/dL). Other co-infections were excluded through a repeat GABHS swab, which was negative, and a multiplex PCR respiratory panel performed on a nasopharyngeal aspirate.

**Figure 2:**
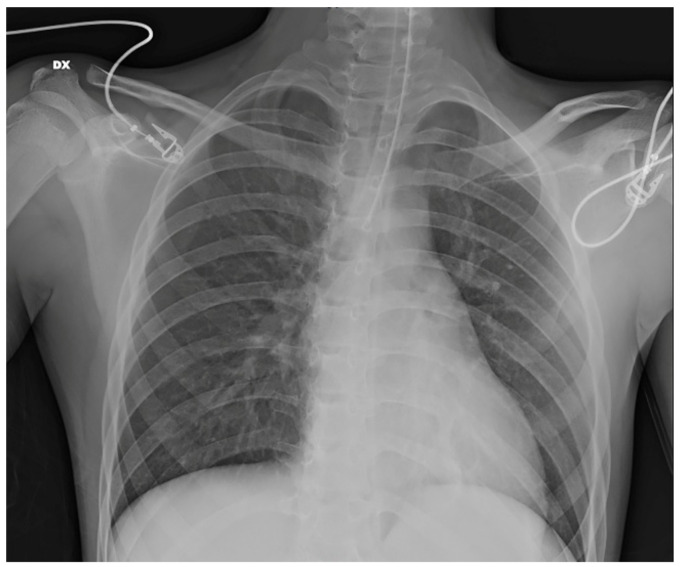
Case 2. Bilateral accentuation of the hilar and perihilar bronchovascular markings. Endotracheal tube tip projected at the D4–D5 level, approximately 1 cm above the carina. DX = right.

Given clinical suspicion of pneumonia, a chest X-ray was performed, showing bilateral accentuation of the hilar and perihilar bronchovascular markings, with a tendency toward confluence in the right upper and lower perihilar regions (Figure 2). An abdominal ultrasound also revealed mild splenomegaly (maximum diameter: 13 cm).

Treatment with high-dose corticosteroids and amoxicillin/clavulanic acid at 75 mg/kg/day was continued.

**Figure 3:**
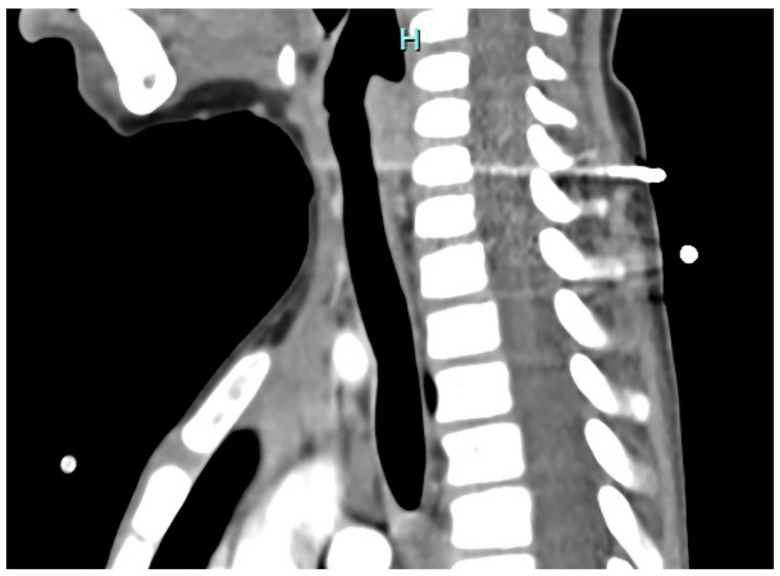
Case 2. Neck and chest CT scan showing preserved tracheal patency. H = upper.

Seventy-two hours after intubation, the first extubation attempt was unsuccessful. A second attempt, performed 48 h later with ENT assistance, resulted in successful extubation under video-laryngoscopic guidance. During the procedure, a video laryngo-tracheoscopy was conducted under spontaneous breathing, revealing a bulging of the posterior tracheal wall suggestive of mild, localized tracheomalacia of the pars membranacea. Despite this finding, the patient’s recovery was uneventful. A subsequent neck and chest CT scan confirmed preserved tracheal patency with no evidence of obstruction or structural compromise (Figure 3).

Progressive clinical and laboratory improvement followed. The child was discharged 20 days after admission, and an elective extracapsular tonsillectomy was successfully and eventfully performed a few months later.

### 2.3. Case Report 3

The third case involves a 14-year-old, previously healthy, female who presented at our pED in October 2024 with general asthenia, fever, and acute pharyngotonsillitis. She had been treated for a few days with amoxicillin/clavulanic acid prior to admission. Initial blood assessment revealed elevated liver enzymes (ALT: 298 U/L, AST: 380 U/L) and direct hyperbilirubinemia (4 mg/dL), while white blood cell count (with mild monocytosis) and CRP were within normal limits. EBV infection was confirmed by serology and a high viral load (344,800 copies/mL in blood).

The patient was admitted to the pediatric ward. Over the following days, her general condition and laboratory profile progressively worsened, with the development of dysphagia and signs of cholestatic hepatitis. The initial ENT evaluation revealed a severely ill appearance, grade IV “kissing” tonsils with marked adenoid hypertrophy, complete activation of Waldeyer’s ring with friable, bleeding lymphatic tissue, and multiple painful lymphadenopathies.

A broad-spectrum antibiotic regimen was initiated with clindamycin (600 mg three times daily) and piperacillin/tazobactam (4.5 g four times daily), along with high-dose methylprednisolone (initially 30 mg twice daily, increased to 40 mg three times daily). Despite therapy, the patient showed no clinical improvement; fever persisted, and laboratory parameters continued to worsen. New findings included hyperferritinemia (1485–2636 mg/L) and cytopenia (hemoglobin 8.1 g/dL; platelets: 72 × 10^9^/L), raising strong clinical suspicion for EBV-associated hemophagocytic lymphohistiocytosis (EBV-HLH).

As her respiratory function deteriorated and non-invasive ventilation became insufficient to maintain adequate oxygenation, the patient was transferred to the pICU, and endotracheal intubation was performed. To exclude EBV-driven lymphoproliferative disease, an ultrasound-guided cervical lymph node needle biopsy was obtained. Histopathology confirmed a lymphoma-like reactive lymphoid proliferation, consistent with infectious mononucleosis, according to the World Health Organization’s 2016 classification and the International Collaboration on Cancer Reporting’s 2022 refined criteria [9].

Cyclosporin therapy was initiated one week after hospitalization at a dose of 2 mg/kg/day administered as a continuous intravenous infusion, with blood concentrations monitored to maintain trough levels of approximately 150–200 ng/mL. A contrast-enhanced CT scan of the neck and chest ruled out any suppurative complication. However, due to persistent bulky, hyperactivated lymphoid tissue in the upper airways that precluded safe extubation, a temporary tracheostomy was performed.

**Figure 4:**
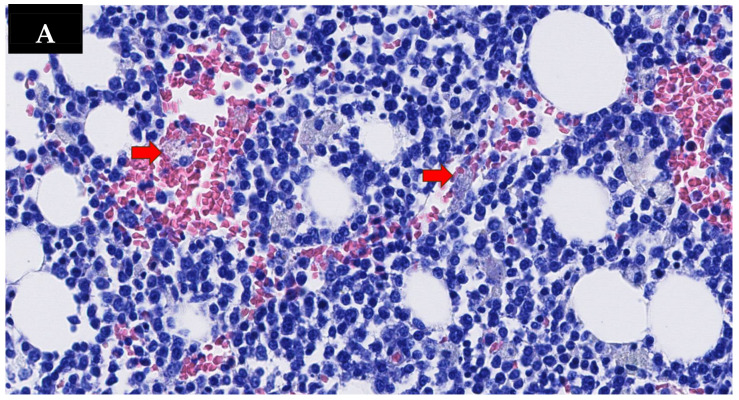

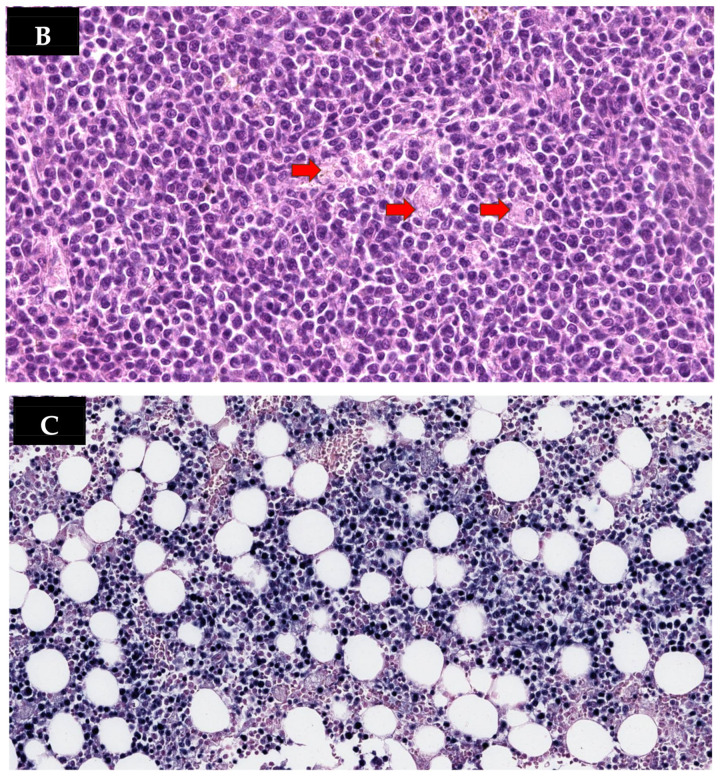
Case 3. Hemophagoytosys in the bone marrow site indicated by arrows (**A**); tissue replacement by polymorphic lymphoid cells, predominantly lymphoplasmacytic, with some large cells, and mixed histiocytes showing cytophagocytosis indicated by arrows (**B**); diffuse nuclear positivity for EBV/EBER at in situ hybridization, blue labeling, confirming a picture of EBV+ polymorphic, destructive B-cell lymphoproliferative disorder (**C**).

Despite intensive care measures and immunosuppressive therapy, the patient’s condition deteriorated further. Three weeks after hospitalization, she developed sepsis and multi-organ failure, which led to death. Autopsy examination confirmed the presence of diffuse multiorgan hemophagocytosis, most evident in the bone marrow, associated with a destructive EBV-positive B-cell lymphoproliferative disorder. Figure 4 documents these histological findings in the liver and bone marrow.

The final EBV viral load measurement showed a markedly elevated level of 62,178,000 copies/mL in blood.

### 2.4. Case Report 4

In March 2025, a 16-year-old, previously healthy male with a recent diagnosis of EBV-related pharyngotonsillitis presented to the pED with neck pain, pharyngodynia, and intermittent fever persisting for 10 days. ENT evaluation revealed painful limitation of neck movement with mild swelling in the upper left laterocervical region, grade II tonsillar hypertrophy, and a left-sided retrotonsillar bulging. Video-laryngoscopy demonstrated swelling of the left oropharynx.

**Figure 5:**
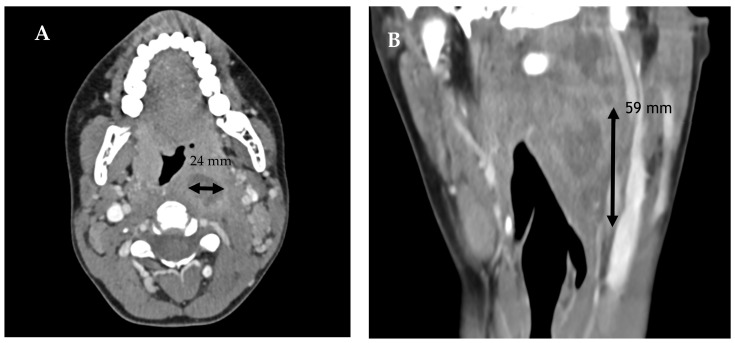
Case 4. Axial (**A**) and coronal (**B**) contrast-enhanced CT scans show a left retropharyngeal abscess, with multiple coalescent liquefactive areas, measuring 16 × 24 mm on the axial plane and 59 mm craniocaudally, protruding into the oropharyngeal lumen.

Given the clinical suspicion of a retropharyngeal abscess, a contrast-enhanced neck CT scan was performed. Imaging revealed swelling in the paramedian retropharyngeal space with a hypodense, non-homogeneous central area measuring approximately 16 × 24 mm on the axial plane and 59 mm in the craniocaudal direction. The mass protruded into the airway, displacing the left palatine tonsil anteromedially and extending toward the superficial neck, compressing the jugular vein (Figure 5).

Laboratory findings initially showed weak positivity on the Monospot test. Subsequently, EBV infection was confirmed by elevated EBV viral capsid antigen (VCA) IgM levels (123 U/mL). Other blood assessments revealed leukocytosis (21.20 × 10^9^/L) with marked neutrophilia (17.53 × 10^9^/L) and monocytosis (2.07 × 10^9^/L), along with a significantly elevated CRP level (255.9 mg/L).

Based on these findings, the patient underwent surgical drainage of the abscess via a transoral approach. Access to the most cranial portion of the collection was achieved through a vertical incision on the left oropharyngeal wall, just posterior to the ipsilateral tonsillar lodge, allowing evacuation of a large volume of purulent secretions.

Following infectious disease consultation, empiric antibiotic therapy with ceftriaxone (2 g/day) and clindamycin (600 mg every 8 h) was initiated. Subsequent culture of the purulent material isolated multi-sensitive *Arcanobacterium haemolyticum*, allowing simplification of the antibiotic regimen to ceftriaxone monotherapy.

The patient’s clinical condition and laboratory parameters progressively improved. A follow-up CT scan performed seven days after surgery showed near-complete resolution of the previously noted hypodense areas of liquefaction. The patient was discharged 11 days after admission, with a recommendation to undergo elective tonsillectomy in the near future.

## 3. Discussion

We present four remarkable cases of EBV-related acute pharyngotonsillitis complicated by life-threatening conditions, such as respiratory distress, EBV-associated hemophagocytic lymphohistiocytosis (EBV-HLH), and retropharyngeal abscess, occurring in children. We selected these cases to draw attention to the potential fatal complications of EBV infection, which, although rare, must be promptly recognized and treated to avoid a poor prognosis. Given the rarity of these complications, there is no clear consensus on treatment strategies.

We specifically selected the most severe cases managed among all children hospitalized between January 2022 and April 2025 in the pediatric ENT, high- or medium-intensity pediatric departments of our tertiary-level pediatric hospital in central Milan for severe IM.

Although EBV-related pharyngotonsillitis during the course of IM is common and generally follows a benign, self-limiting course, in rare cases it may be associated with more severe clinical manifestations and an unfavorable outcome. Such patients are often managed individually by the pediatrician or, sometimes, by the otolaryngologist alone. However, in selected cases, prognosis can be optimized through a multidisciplinary team approach, involving pediatricians, otolaryngologists, infectious disease specialists, radiologists, and intensive care specialists.

Acute airway obstruction in patients with IM is rare, with an incidence reported between 1–3.5% [10,11,12]. This complication may present suddenly and requires prompt otolaryngologic and anesthesiologic support. In some instances, activation of pharyngeal lymphatic tissue is so florid and abrupt that even high-dose systemic steroid treatment is insufficient to reduce the swelling quickly. When it occurs, appropriate airway management is essential, and in severe cases, endotracheal intubation or emergency tracheostomy may be required. The incidence of serious acute upper airway obstruction during primary EBV infection increases with age [13]. Respiratory symptoms and fever >10 days should be considered “red flags” for life-threatening complications [14]. Early diagnosis and prompt intervention are crucial, as there are no universally accepted criteria distinguishing conservatively managed cases from those requiring surgery.

Tonsillar enlargement occupying more than 75% of the oropharyngeal inlet can rapidly lead to severe airway compromise, even though the patient may initially appear deceptively calm [15]. This progression is more common in younger children, where respiratory distress can develop quickly due to the relatively small size of the oro-laryngopharyngeal space in comparison to enlarged tonsils. In such cases, the presence of anesthesiologists experienced in pediatric care and access to a pediatric intensive or sub-intensive care unit are mandatory to ensure safe management. Given the unpredictability of this evolution, multidisciplinary collaboration between ENT specialists, pediatricians, and anesthesiologists from patient arrival is highly desirable, especially in cases at greatest risk for complications.

When significant tonsillar hypertrophy results in upper airway obstruction, urgent tonsillectomy may be required to restore oropharyngeal patency. However, the increased risk of primary and secondary bleeding due to acute, florid inflammation should be considered. In this context, intracapsular tonsillectomy (tonsillotomy) may be preferred over traditional extracapsular tonsillectomy during the acute phase, as it carries a lower—though not negligible—risk of hemorrhage [13]. In our first patient, intracapsular tonsillectomy enabled successful extubation, although two post-operative bleeding episodes occurred, each requiring surgical revision.

Historically, tonsillectomy has been suggested to shorten the clinical course in patients with IM presenting with marked tonsillar involvement, dating back from the 1930s to the 1970s. Case reports, retrospective studies, and expert commentaries described improved clinical outcomes following tonsillectomy, with earlier recovery and no increase in complication rates [16]. Nevertheless, these studies were neither randomized nor included control groups, and no randomized controlled trials on the role of tonsillectomy in IM were published between 1984 and 2014. Clinical practice guidelines from the USA, France, and other countries do not list IM as an indication for tonsillectomy.

According to Windfuhr et al. [6], tonsillectomy should not be routinely performed for symptom control or to shorten disease course. However, it may be considered in selected patients with clinically significant upper airway obstruction caused by severe inflammatory tonsillar hyperplasia. When deciding between tonsillectomy and tonsillotomy, the surgical approach should aim to minimize hemorrhage risk. The literature reports varying rates of postoperative bleeding; for example, Lloyd [14] described intracapsular tonsillectomy (tonsillotomy) cases without postoperative bleeding, symptom recurrence, or readmission. In contrast, older studies advocated acute tonsillectomy to relieve airway obstruction unresponsive to corticosteroids [17], though this approach is not widely adopted due to perioperative bleeding rates as high as 13% [18].

Our third case compelled ENT and pediatric teams to confront a rare EBV complication—EBV-HLH. This syndrome is characterized by fever, pancytopenia, organomegaly, multiorgan dysfunction, and hemophagocytosis mediated by excessive activated macrophages infiltrating bone marrow and other tissues. EBV is the most frequently reported infectious trigger for secondary HLH [19,20]. Diagnostic guidelines for HLH [21] identify eight criteria, five of which must be met for diagnosis: persistent fever; splenomegaly; cytopenias affecting ≥2 lineages; hypertriglyceridemia and/or hypofibrinogenemia; evidence of hemophagocytosis; low/absent NK cell activity; hyperferritinemia; and elevated soluble IL-2 receptor (sIL-2r) levels. Ferritin > 500 µg/L is a particularly valuable biomarker.

Recent studies report variable prognosis: overall survival (OS) ranges from weeks to several years. Some children achieve long-term remission with immunotherapy or chemotherapy; others may die within months, even after allo-HSCT [22,23]. The HLH-2004/94 protocol (etoposide + dexamethasone) remains standard of care [24], with selective allo-HSCT for primary HLH or refractory cases [25]. EBV-HLH secondary to IM in adolescents is extremely rare, with an estimated incidence of 1 in 800,000 individuals/year [26,27]. Non-response to first-line therapy is associated with poor prognosis and rapid clinical deterioration.

The final case describes a retropharyngeal abscess as a rare EBV complication. EBV-induced transient immunosuppression, particularly reduced T-cell-mediated immunity, predisposes patients to secondary bacterial infections, allowing commensal oral flora to become pathogenic. In our patient, the abscess extended between the alar and prevertebral fascia, providing a direct route to the mediastinum and pleural spaces, potentially causing descending necrotizing mediastinitis—a life-threatening condition [28,29,30].

Two main mechanisms for mediastinitis have been described: direct extension through fascial planes or hematogenous spread via septic thrombophlebitis, as in Lemierre’s syndrome [31]. In our case, the likely pathogenetic mechanism was retropharyngeal reactive lymphadenitis with abscess formation.

For deep neck space infections, surgical options include needle aspiration, incision and drainage, or abscess tonsillectomy. Oral approaches are preferred when feasible, but airway patency must be carefully evaluated. Intubation may be technically challenging or risky due to laryngeal edema or abscess protrusion, and emergency tracheotomy may be necessary. Postoperative monitoring should continue for ≥48 h.

In life-threatening retropharyngeal abscess, elective tonsillectomy is indicated after acute resolution. Routine tonsillectomy for symptom control in mononucleosis is not recommended, whereas tonsillotomy is indicated in acute cases with airway obstruction due to inflammatory tonsillar hyperplasia [32]

This case series expands on the existing literature by presenting detailed clinical descriptions of severe ENT-related complications of pediatric acute IM, including life-threatening airway obstruction, HLH, and retropharyngeal abscess—complications rarely documented in previous reports—thereby highlighting the spectrum of potential severe outcomes and the critical importance of early recognition and multidisciplinary management. Although some of these complications have been previously reported in the literature, often as isolated or even sporadic cases, there is a paucity of case series providing a broader perspective on the spectrum of ENT-related complications in pediatric acute IM presenting predominantly with early-stage pharyngotonsillar involvement. This series documents multiple, diverse severe complications within the same pediatric population, offering a more comprehensive understanding of potential clinical presentations and emphasizing the importance of early recognition and multidisciplinary management.

## 4. Conclusions

The prognosis for IM in the pediatric population is generally favorable, although acute complications may occur. Severe complications are rare, and most resolve spontaneously. However, life-threatening cases may result from uncontrolled lymphoproliferative responses that compromise immunity. These case reports highlight the diagnostic challenges faced by ENT specialists. ENT teams play a crucial role in identifying when EBV-related tonsillitis may progress to severe sequelae. The systemic effects of mononucleosis must be considered, and surgical intervention should be carefully evaluated, particularly in cases of airway obstruction or refractory superinfections.

Multidisciplinary collaboration with pediatricians and pediatric intensivists is essential. Decisions regarding invasive, high-risk procedures must carefully weigh risks and benefits, relying on sound clinical judgment.

Due to the rarity of these emergent presentations, the literature lacks standardized guidelines for managing life-threatening EBV-related tonsillitis in children. Cases are typically managed on an individual basis, depending on the experience and expertise of the treating team. There is a clear need for further research and the development of evidence-based protocols to guide optimal care in these complex scenarios.

## Figures and Tables

**Table 1:** Clinical data of patients admitted in hospital with severe IM.

Case Report	1	2	3	4
Age at diagnosis, Gender	14 y, F	5 y, M	14 y, F	16 y, M
Symptoms	Persistent pharyngodynia and fever despite first-line antibiotic therapy	Fever and sore throat, with progressive dyspnea and deterioration of overall clinical condition	General asthenia, fever and acute pharyngotonsillitis, with progressive dysphagia and dyspnea	Neck pain, pharyngodynia, and intermittent fever persisting for 10 days
Laboratory findings	EBV viral capsid antigen (VCA) IgM levels > 160 U/mL, elevated liver enzymes, and leucocytosis with lymphomonocytosis	EBV infection, with a high viral load (97,393 EBV copies/mL in blood and 340,000 copies/mL in the tonsillar swab), positive EBV serology (EBV-VCA IgM 129 U/mL), and elevated CRP levels (1.20 mg/dL)	At admission, elevated liver enzymes (ALT 298 U/L, AST 380 U/L), direct hyperbilirubinemia (4 mg/dL), mild monocytosis, normal CRP, and EBV infection confirmed by serology with a high viral load (344,800 copies/mL in blood). The clinical course progressively worsened with hyperferritinemia (1485–2636 mg/L) and cytopenia (hemoglobin 8.1 g/dL; platelets 72 × 10^9^/L)	Initial weak positivity on the Monospot test; subsequent confirmation of EBV infection by elevated EBV viral capsid antigen (VCA) IgM levels (123 U/mL); leukocytosis (21.20 × 10^9^/L) with marked neutrophilia (17.53 × 10^9^/L), monocytosis (2.07 × 10^9^/L), elevated CRP level (255.9 mg/L)
Radiological exams	Neck and abdominal ultrasound, contrast- enhanced neck CT scan, and brain MRI due to persistent neurological slowing and aphasia of uncertain origin	Chest X-ray, complete abdominal ultrasound, and neck and chest CT scan	Ultrasound-guided cervical lymph node needle biopsy, and CT scans of the head and neck and abdomen	Neck CT scan
Complications	Cardiorespiratory arrest during a neck ultrasound, requiring emergency intubation, followed by multiple surgical procedures (initially a tonsillotomy, then a complete tonsillectomy due to episodes of bleeding)	Intubation, and one failed attempt of extubation	Need for invasive mechanical ventilation requiring endotracheal intubation followed by tracheostomy, vasopressor support due to progressive organ failure and sepsis, and final histopatological confirmation of a lymphoma-like reactive lymphoid proliferation	Surgical drainage of a left para-retropharyngeal abscess
Outcome	Post-traumatic stress disorder, with complete recovery achieved nine months after hospital discharge	Progressive clinical and laboratory improvement, the patient was discharged after 20 days, and extracapsular tonsillectomy was performed a few months later	Death occurred three weeks after hospitalization	Full recovery achieved with targeted antibiotic therapy; the patient was discharged after 11 days, and tonsillectomy was recommended subsequently

## Data Availability

Data available on request due to privacy/ethical restrictions.

## Abbreviations

The following abbreviations are used in this manuscript:

IM	Infectious mononucleosis
EBV	Epstein-Barr Virus
ENT	Ear, nose, throat
pED	Pediatric emergency department
VCA	Anti-viral capsid antigen
AST	Aspartate transaminase
ALT	Alanine aminotransferase
GGT	Gamma-glutamyltransferase
LDH	Lactate dehydrogenase
pICU	Pediatric intensive care unit
CT	Computed tomography
SBEGA	Group A beta-hemolytic streptococcus
CRP	C-reactive protein
HLH	Hemophagocytic lymphohistiocytosis
OS	Overall survival
